# The Effects of *Chlorella vulgaris* on Polycystic Ovary Syndrome in Mice

**DOI:** 10.1002/fsn3.70992

**Published:** 2025-10-15

**Authors:** Yu Sun, Yanan Wang, Jiaojiao Qi, Ruipeng Gao, Suxu Tan, Shuang Wang, Qing Zhang, Xuelin Gong, Shichao Xing, Zhenxia Sha

**Affiliations:** ^1^ Institute of Aquatic Biotechnology, College of Life Sciences Qingdao University Qingdao Shandong China; ^2^ Shandong Center of Technology Innovation for Biological Breeding of Premium Fish (Preparatory) Yantai Shandong China; ^3^ Qingdao Women and Children's Hospital Qingdao Shandong China

**Keywords:** *Chlorella*, gut microbiota, oxidative stress, polycystic ovary syndrome, steroid hormones

## Abstract

This study aimed to investigate the therapeutic effects of *Chlorella* on ovarian hormone levels, steroidogenic enzymes, ovarian dysfunction, oxidative stress, and gut microbiota composition in a mouse model of polycystic ovary syndrome (PCOS). A PCOS mouse model was established using dehydroepiandrosterone (DHEA) induction. The effects of *Chlorella* supplementation were evaluated in vivo to determine its potential for alleviating PCOS‐related symptoms. *Chlorella* treatment effectively reduced testosterone (T) and luteinizing hormone (LH) levels in mice with PCOS. Additionally, *Chlorella* modulated the expression of oxidative stress‐related genes and improved ovarian morphology and function. Notably, *Chlorella* also restored the gut microbiota from PCOS‐associated dysbiosis. *Chlorella* ameliorated PCOS symptoms by rebalancing ovarian steroid hormones, enhancing antioxidant defense, and modulating the gut microbiota. These findings support its potential as a natural therapeutic agent for PCOS management.

## Graphical abstract

A mouse model of polycystic ovary syndrome was successfully induced in our experiments. Therapeutic effects of chlorella algal powder as a functional food to some extent in a mouse model of polycystic ovary syndrome.

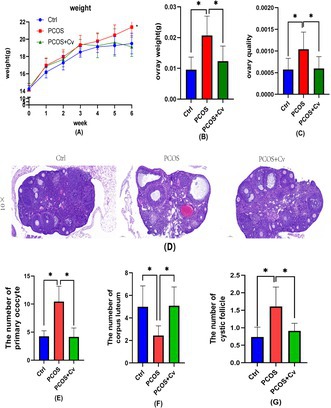

## 1 Introduction

Polycystic ovary syndrome (PCOS) is a prevalent endocrine‐metabolic disorder affecting women of reproductive age, with a global prevalence estimated at 10%–13% (Hong et al. 2023; Valdimarsdottir et al. 2025). It is primarily characterized by hyperandrogenism, ovulatory dysfunction, and polycystic ovarian morphology (Goodarzi et al. 2011). PCOS is commonly associated with insulin resistance (Gao, Zhao, et al. 2024; Zhang et al. 2024), obesity, cardiovascular diseases (Hetherington et al. 2024; Pililis et al. 2024), and infertility (Tan et al. 2024), all of which significantly impair women's health and quality of life. Despite extensive research, the underlying pathophysiology of PCOS remains unclear (Han et al. 2024). Current clinical treatments, including short‐acting contraceptives, metformin, and other pharmacological agents, are limited by their side effects and do not fundamentally alter the disease's progression (Aksun et al. 2025; Li et al. 2019; Patel 2018). Therefore, it is imperative to explore safer and more effective alternative therapeutic strategies.

In recent years, natural products have gained increasing attention as potential interventions for PCOS due to their multi‐target regulatory properties and relatively low toxicity. *Chlorella vulgaris* (Cv), a unicellular green microalga rich in proteins, polysaccharides, chlorophyll, and other bioactive compounds, has demonstrated multiple health benefits, including antioxidant, anti‐inflammatory, lipid‐regulating, and insulin‐sensitizing effects (Ji and Fan 2020; Cho et al. 2024; Melo et al. 2024; Silva et al. 2025). For example, *Chlorella*‐derived polysaccharides have been shown to reduce blood lipid levels by activating the AMPK signaling pathway (Chen et al. 2021), while chlorophyll derivatives can alleviate oxidative stress‐induced damage (Fiorentino et al. 2025). Given the multifactorial etiology of PCOS, involving metabolic disturbances, chronic inflammation, and oxidative stress, the diverse bioactivities of *Chlorella* may offer a novel and promising approach for PCOS management (Orisaka et al. 2023). However, limited studies have investigated the therapeutic efficacy and underlying mechanisms of *Chlorella* in the context of PCOS.

In this study, we established a dehydroepiandrosterone (DHEA)‐induced PCOS mouse model to comprehensively evaluate the effects of *Chlorella* on endocrine disturbances, ovarian dysfunction, and metabolic abnormalities. Additionally, we explored the potential molecular mechanisms, aiming to provide a scientific basis for the development of natural‐product‐based interventions for PCOS.

## 2 Materials and Methods

### 2.1 Animals

Female C57BL/6J mice (21 days old) were purchased from Jinan Pengyue Biotechnology Co. Ltd. The rearing conditions are as follows: temperature 24°C, humidity 50%, light for 12 h (08:00–20:00), darkness for 12 h (20:00–08:00 the next day), with free access to food and water. After 1 week of acclimatization, the animals were randomly assigned to three groups (*n* = 10 per group): control (Ctrl), polycystic ovary syndrome model (PCOS), and *Chlorella*‐treated PCOS (PCOS + Cv). To establish the PCOS model, mice in the PCOS and PCOS + Cv groups received daily subcutaneous injections of 0.1 mL DHEA at a dose of 60 mg/kg, dissolved in 95% ethanol with 10% sesame oil, for 21 consecutive days (Gao, Wang, et al. 2024; Yu et al. 2023). The control group received equivalent volumes of solvent without DHEA.

Following modeling, mice in the PCOS + Cv group were administered wall‐broken *Chlorella* suspension at a dose of 400 mg/kg by oral gavage. At the end of the sixth week, all animals were anesthetized using isoflurane and sacrificed. Blood and ovarian tissues were collected for further analysis.

All animal procedures were approved by the Animal Research Ethics Committee of the Affiliated Hospital of Qingdao University (Approval code: AHQU‐MAL20230602) and conducted in accordance with the ARRIVE guidelines.

### 2.2 Cell Line

Human ovarian granulosa‐like tumor cells (KGN) were obtained from the Shanghai Cell Bank (Chinese Academy of Sciences). Cells were cultured in DMEM/F12 medium (Procell Life Sciences Co. Ltd.) supplemented with 5% horse serum and 2.5% fetal bovine serum (Procell Life Sciences Co. Ltd.). KGN cells were divided into three groups: Ctrl, PCOS, and sulforaphane (SFN). Sulforaphane, a well‐known Nrf2 activator, was used in this study to verify the function of the Keap1/Nrf2 pathway in oxidative stress. The PCOS model was established by treating cells with 10 μM DHEA, while the SFN group was treated with both 10 μM DHEA and 10 μM SFN. After 24 h of treatment, total RNA was extracted for subsequent analysis, as described previously (Gao, Wang, et al. 2024).

### 2.3 Biochemical Parameter Analysis

Serum concentrations of hormones including testosterone (T), gonadotropin‐releasing hormone (GnRH), luteinizing hormone (LH), follicle‐stimulating hormone (FSH), and progesterone (P) were measured using ELISA kits (MEIMIAN, Jiangsu, China). Levels of malondialdehyde (MDA) and glutathione (GSH) in mouse serum, ovarian tissues, and cell supernatants were determined using commercial kits (Nanjing Jiancheng Bioengineering Institute, Jiangsu, China). In addition, the concentration of cellular reactive oxygen species (ROS) was determined using commercially available kits (Elabscience Biotechnology Co. Ltd).

### 2.4 Morphological Observation of Ovaries

The bilateral ovaries of mice were rapidly excised, fixed in 4% paraformaldehyde, and embedded in paraffin for routine histological processing. Tissue sections (6 μm thick) were prepared and stained with hematoxylin and eosin (H&E). Ovarian morphology and structural features were examined and photographed under a light microscope, and follicles at all developmental stages were quantitatively assessed.

### 2.5 Fluorescence Quantitative PCR

Total RNA extracted in the previous steps was inverted according to the All‐In‐One 5X RT MasterMix. The sample was transcribed into complementary DNA (cDNA) and analyzed using a Roche LightCycler 96 fluorescent quantitative PCR instrument. The reaction utilized FS Universal SYBR Green Real Master fluorescent quantitative PCR dye (Roche). The reaction system for PCR was 10 μM upstream primer, 10 μM downstream primer, 0.25 μL each, 1 μL of cDNA template, 5 μL of Blas TaqTM 2× qPCR, and 10 μL dH2O of to make up the reaction system.

PCR reaction conditions (two‐step method): pre‐denaturation at 95°C for 3 min; denaturation at 95°C for 15 s; followed by annealing and extension at 60°C for 1 min, followed by 40–50 cycles.

At the end of the reaction, the results were analyzed using the software provided with the fluorescence quantitative PCR instrument. The 2^−ΔΔ*Ct*^ method was employed to interpret the reaction outcomes. The gene expression levels were calculated using the method, with glyceraldehyde‐3‐phosphate dehydrogenase (GAPDH) serving as the internal reference gene. The prism list is in Table S1.

### 2.6 Western Blotting Analysis

For western blot experiments, proteins were first extracted from the ovaries using RIPA reagent. The protein‐containing lysates were centrifuged and then quantified using BCA reagent. Then, 30 μg of proteins were separated by electrophoresis on 10% SDS‐PAGE and then loaded onto a PVDF membrane and incubated in 25% skimmed milk for 120 min. The membrane was then incubated with a primary antibody solution targeting the indicated proteins. The antibodies used included monoclonal antibodies against NRF2, GPX4, KEAP1, STAR, CYP11A1, CYP19A1, and an anti‐β‐Actin antibody. After incubation, the membrane was washed three times with PBST and incubated with secondary antibody solution at room temperature for 120 min. The secondary antibody was recovered, and the membrane was washed three times with PBST. Prepare the development solution according to the instructions, add it dropwise on the membrane, put it into the chemiluminescence developer, and take pictures for storage. Finally, the bands were analyzed by densitometry using ImageJ software.

### 2.7 Fecal Collection and 16S rRNA Sequencing

Fresh fecal samples were collected from each group of mice into sterile EP tubes by 10:00 a.m. during the sixth week of the experiment. Samples were immediately frozen and stored at −80°C until further analysis. The composition of the intestinal microbiota was analyzed using 16S rRNA gene sequencing. Total DNA was extracted from fecal samples, followed by PCR amplification, sequencing, and downstream microbial analysis, as previously described (Xin et al. 2024).

After raw sequencing data were quality‐filtered, sequences with ≥ 97% similarity were clustered into operational taxonomic units (OTUs). These OTUs were taxonomically classified at different phylogenetic levels, with a focus on genus‐level annotations. Alpha diversity was assessed using indices such as Chao1, Sobs, and ACE to evaluate species richness within individual samples. Beta diversity was analyzed using both weighted and unweighted UniFrac distance metrics. Principal coordinate analysis (PCoA) and principal component analysis (PCA) were used to visualize differences in microbial community structure across groups.

### 2.8 Statistical Analysis of Data

All data were first tested for normality. For comparisons between two groups, independent *t*‐tests were performed. One‐way analysis of variance (ANOVA) followed by Tukey's post hoc test was used for multiple group comparisons. For data that did not follow a normal distribution, non‐parametric tests (Kruskal–Wallis test) were applied. Results were presented as mean ± standard deviation (SD). Statistical analyses and graph generation were performed using GraphPad Prism version 9.5. A *p*‐value of < 0.05 was considered statistically significant, and *p* < 0.01 was regarded as highly significant.

## 3 Results

### 3.1 Effects of *Chlorella* Powder on Body Weight and Ovaries of PCOS Mice

**FIGURE 1:**
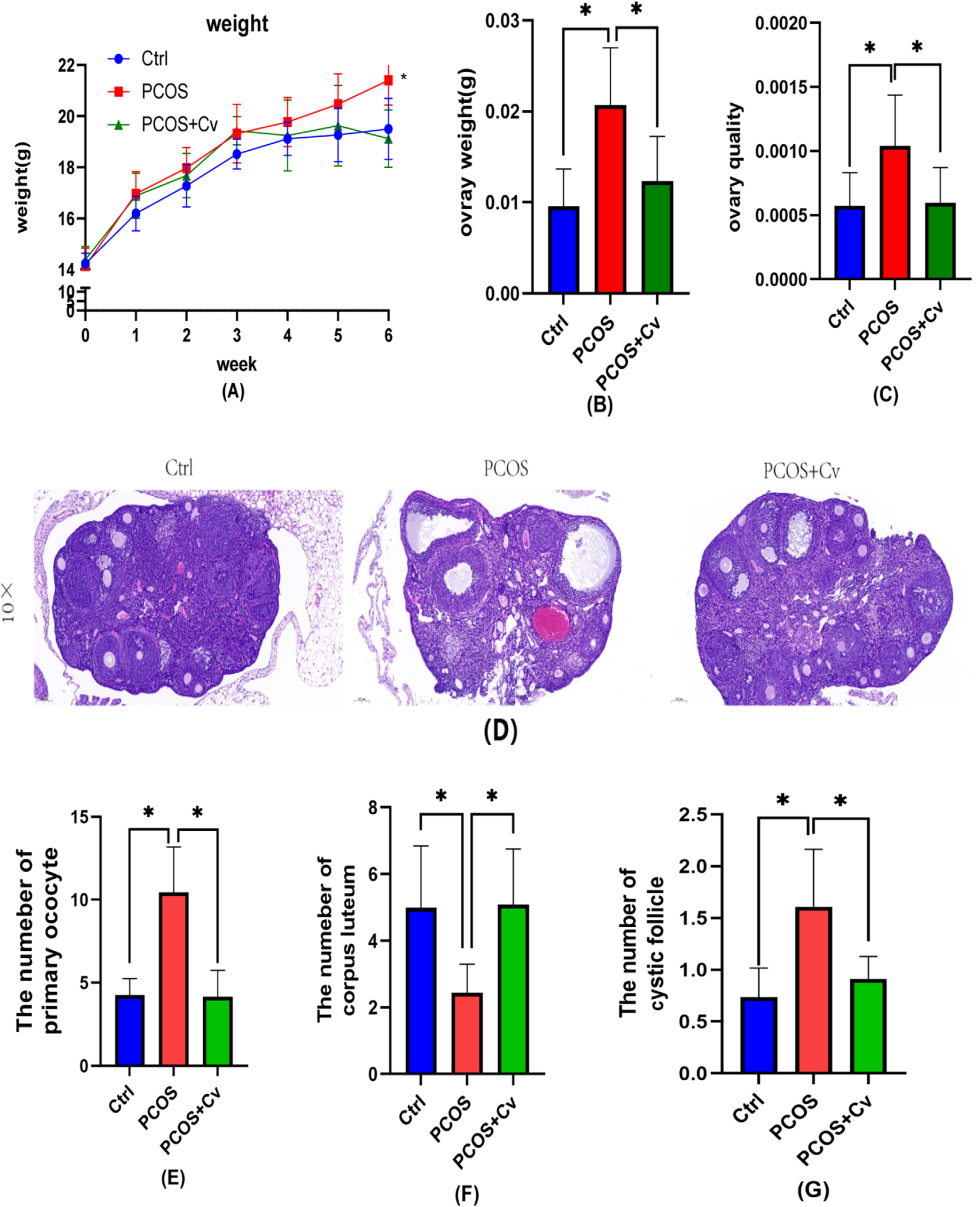
Comparative analysis of body weight, ovarian morphology, and histological features across three experimental groups. (A) Analysis of the body weight (g) of three groups plotted over time (weeks). (B) The weights (g) of the ovaries in the three groups. (C) The ovarian quality for the three groups. (D) Hematoxylin and eosin (H&E) staining. (E–G) Counting of different types of follicles. Figures show 10× magnification. Scale bar = 100 μm for 10×. The exact sample size (*n* = 6) for each experimental group; statistical significance: ns = not significant, *p < 0.05.

To verify the protective effect of *Chlorella* on PCOS mice, we established PCOS model mice and gavaged them with *Chlorella* solution. By week 6, the body weight of the PCOS group was significantly higher than that of the Ctrl and PCOS + Cv groups (Figure 1A). Ovarian weight and ovarian quality were significantly elevated in the PCOS group compared to the Ctrl group, whereas treatment with *Chlorella* resulted in a significant reduction of both (Figure 1B,C). The increased ovarian weight suggested the occurrence of follicular accumulation and mesenchymal hyperplasia in the ovary, while the elevated ovarian quality was also one of the markers of successful establishment of the PCOS model. H&E staining showed that more cystic follicles and a decrease in the number of luteinization were present in the ovaries of the mice in the PCOS group (Figure 1D–G). In contrast, all measured parameters were significantly restored in the PCOS + Cv group compared to the PCOS group. *Chlorella* improves body weight and ovarian status in PCOS model mice when abnormalities occur.

### 3.2 Effect of *Chlorella* Powder on Serum Hormones in PCOS Mice

**FIGURE 2:**
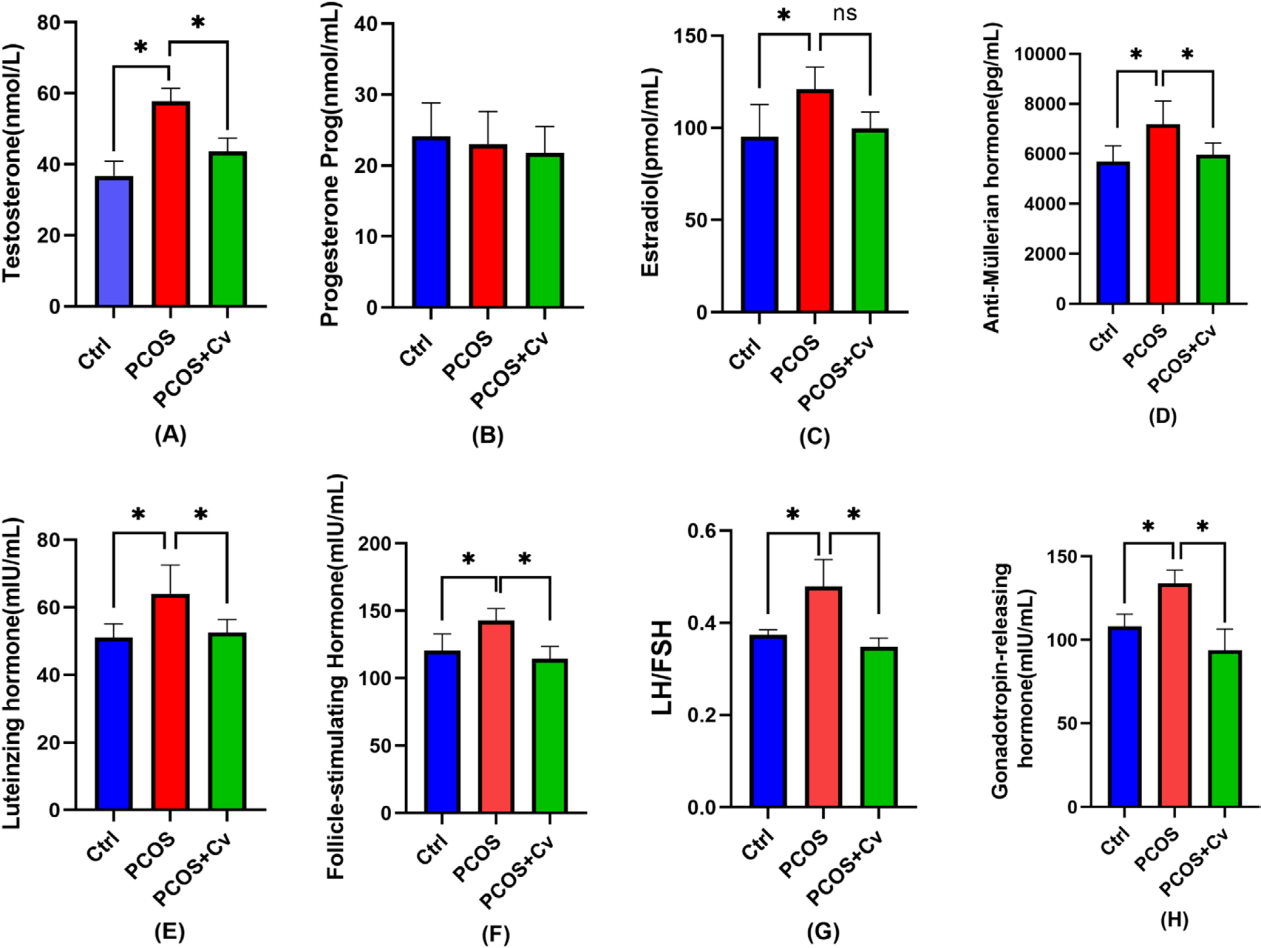
(A–H) Reproductive and hormonal markers including T (A), P (B), E_2_ (C), AMH (D), LH (E), FSH (F), and GnRH (H) in serum of three groups of mice. Data are expressed as mean ± standard deviation, and comparisons between groups were statistically significant. Statistical significance is indicated: ns = not significant, *p < 0.05. The exact sample size (*n* = 6) for each experimental group.

In order to investigate the effects of *Chlorella vulgaris* on the sex hormones of PCOS mice, we examined the levels of serum T, P, E_2_, AMH, LH, FSH, GnRH, and the LH/FSH ratio. As shown in Figure 2, serum T, E_2_, AMH, LH, and GnRH levels were elevated in the PCOS group, while FSH levels were significantly decreased. In contrast, P serum levels remained unchanged (Figure 2B). In the PCOS + Cv group, T, LH, AMH, E_2_, GnRH levels decreased, and LH/FSH returned to normal (Figure 2G). These observations suggest that *Chlorella* modulates serum hormone levels and decreases serum testosterone levels in PCOS mice.

### 3.3 *Chlorella* Regulates the Expression of Ovarian Steroidogenic Enzymes in PCOS Mice

**FIGURE 3:**
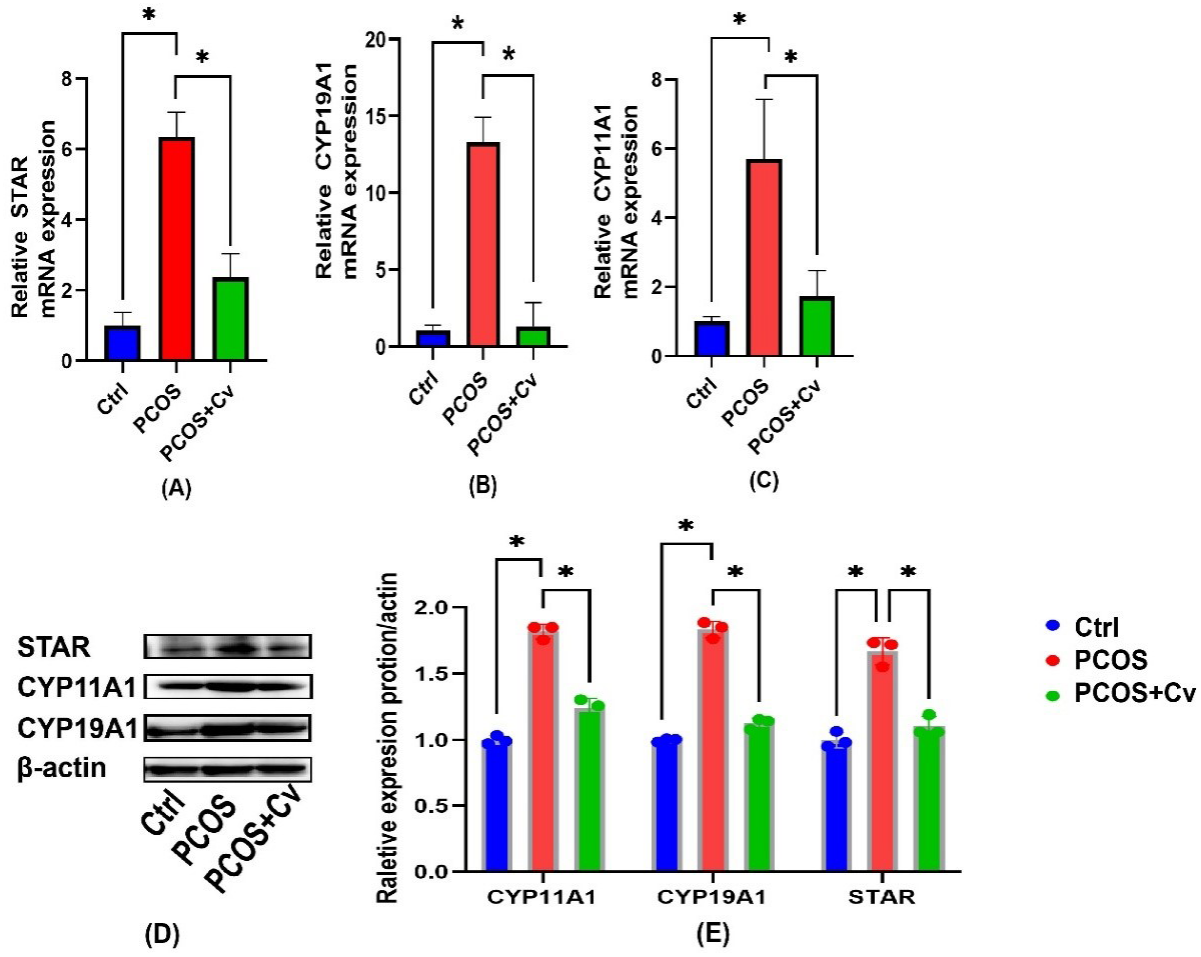
Analysis of key enzymes for hormone synthesis. (A–C) Analysis of mRNA expression of key hormone synthetic enzymes in the ovaries of different groups. (D) Western blot analysis of related proteins in different groups of ovaries. (E) Relative expression of proteins in (E). Statistical significance is indicated: ns = not significant, *p < 0.05. The exact sample size (*n* = 3) for each experimental group.

To further investigate the effects of *Chlorella* on hormone biosynthesis in PCOS mice, we analyzed the expression of key steroidogenic enzymes in ovarian tissues. Both mRNA and protein levels of *STAR, CYP19A1*, and *CYP11A1* were significantly upregulated in the PCOS group compared to the control (Figure 3A–E). In contrast, treatment with *Chlorella* markedly reduced the expression of these genes and proteins in the PCOS + Cv group. These results suggest that *Chlorella* may inhibit excessive testosterone production by downregulating key steroidogenic enzymes in the ovary.

### 3.4 *Chlorella* Reduces Systemic and Ovarian Oxidative Stress

**FIGURE 4:**
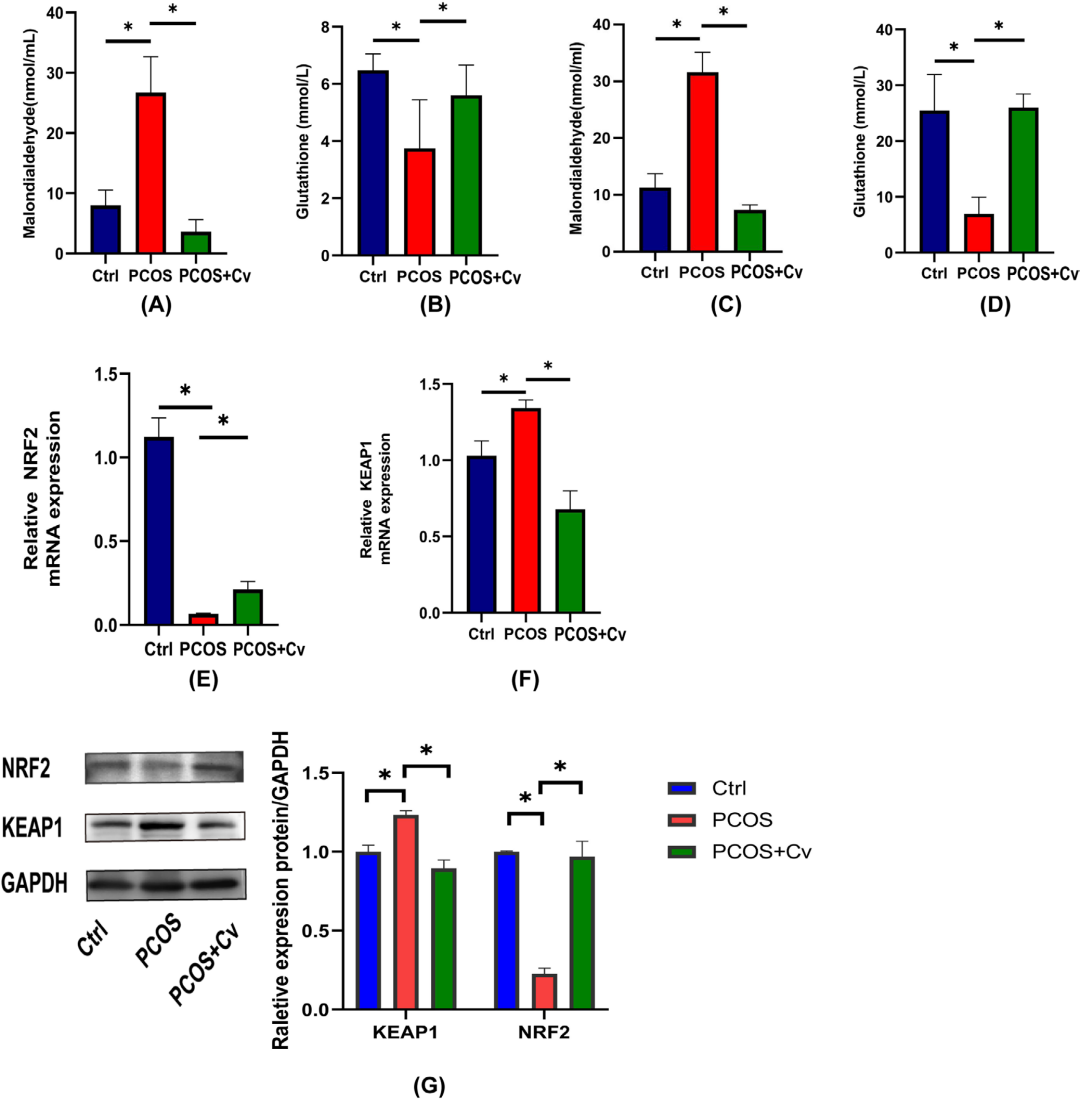
Analysis of oxidative stress in PCOS mice. (A) MDA content in the serum of different groups. (B) Analysis of GSH content in the serum of different groups. (C) MDA content in the ovaries of different groups. (D) Analysis of GSH content in the ovaries of different groups. (E, F) Expression of *KEAP1* and *NRF2* mRNA in the ovaries. (G) Western blotting for detection of KEAP1 and NRF2 protein expression in blot images and quantification. Statistical significance is indicated: ns = not significant, *p < 0.05. The exact sample size (*n* = 3) for each experimental tal group.

To evaluate the role of *Chlorella* in mitigating oxidative stress in PCOS mice, levels of MDA and GSH were measured in both serum and ovarian tissues using commercial assay kits (Figure 4A–D). Compared with the Ctrl group, the PCOS group showed significantly elevated MDA levels and reduced GSH levels (*p* < 0.05), indicating enhanced oxidative damage. Notably, *Chlorella* supplementation in the PCOS + Cv group significantly reduced MDA levels and increased GSH levels, suggesting improved antioxidant status. Given the critical role of the KEAP1/NRF2 signaling pathway in oxidative stress regulation, we further analyzed the expression of related genes and proteins in ovarian tissues using qPCR and Western blot (Figure 4E–H). Compared with PCOS mice, *Chlorella* administration significantly suppressed KEAP1 expression while upregulating NRF2 and GPX4 at both mRNA and protein levels. These results suggest that *Chlorella* alleviates oxidative stress by activating the KEAP1/NRF2/GPX4 pathway in the ovaries of PCOS mice.

**FIGURE 5:**
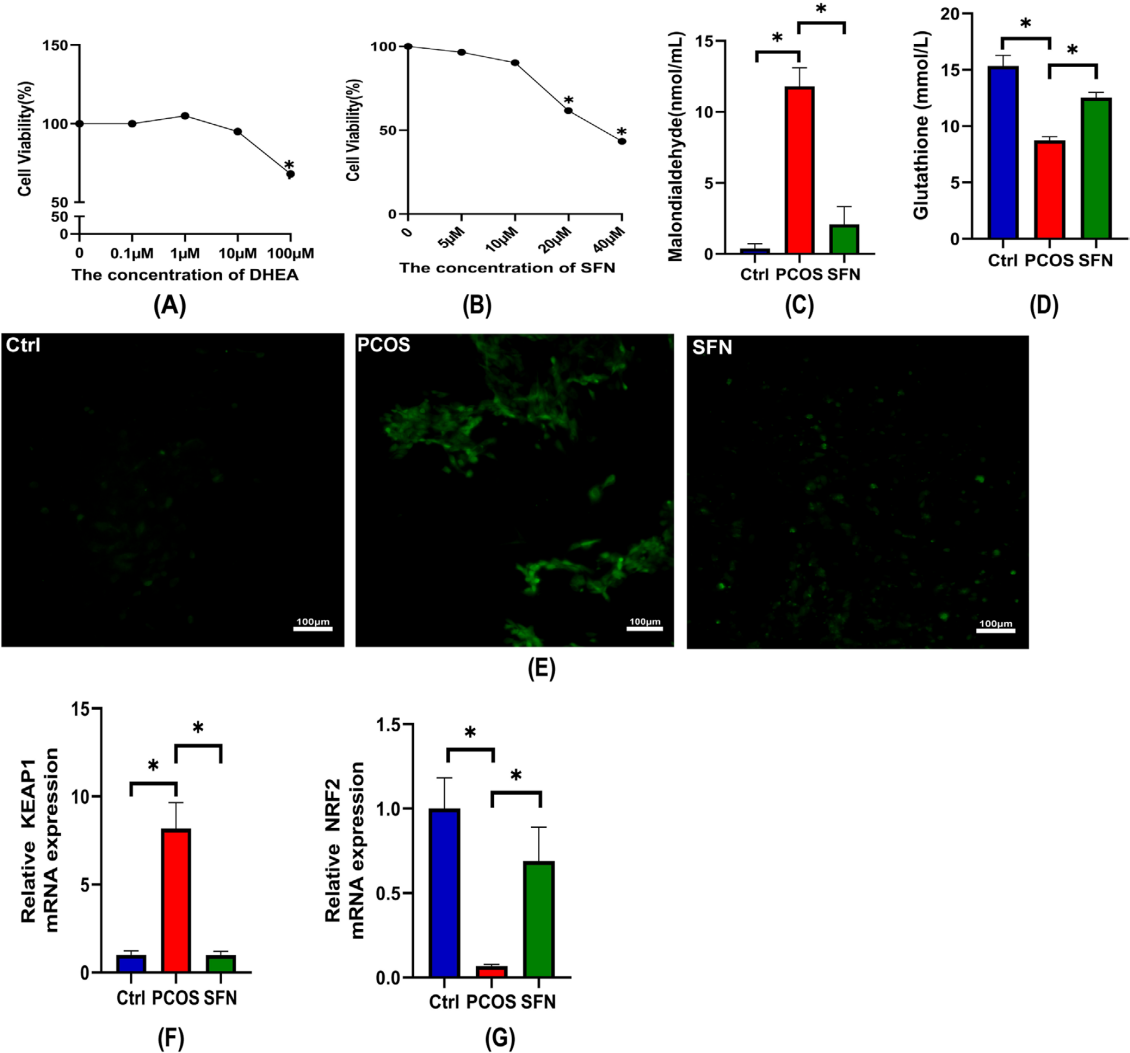
NRF2/KEAP1 function validation. (A) Survival of KGN cells in different concentrations of DHEA. (B) Survival of KGN cells in different concentrations of SFN solution. (C) MDA levels in the supernatant of KGN cells. (D) GSH level in the supernatant of KGN cells. (E) ROS levels under different treatments. (F, G) NRF2, KEAP1 mRNA expression in cells after different treatments. *p < 0.05.

To elucidate the role of the NRF2/KEAP1 signaling pathway in ovarian oxidative stress, a PCOS cell model was established using KGN cells treated with various concentrations of DHEA and SFN for 24 h. As shown in Figure 5A,B, high concentrations of DHEA significantly reduced cell viability, while 10 μM DHEA showed minimal cytotoxicity and was therefore selected for subsequent experiments. Treatment with SFN effectively improved oxidative stress markers. Compared to the Ctrl group, the PCOS group exhibited significantly increased levels of ROS and MDA and decreased GSH levels. SFN supplementation reversed these changes by reducing ROS and MDA levels while increasing GSH content (Figure 5C–E). Additionally, qPCR analysis revealed that SFN significantly downregulated KEAP1 mRNA expression and upregulated NRF2 mRNA expression in KGN cells (Figure 5F–G), suggesting that SFN mitigates oxidative stress by activating the NRF2/KEAP1 pathway.

### 3.5 Effects of *Chlorella* on the Intestinal Flora of PCOS Mice

**FIGURE 6:**
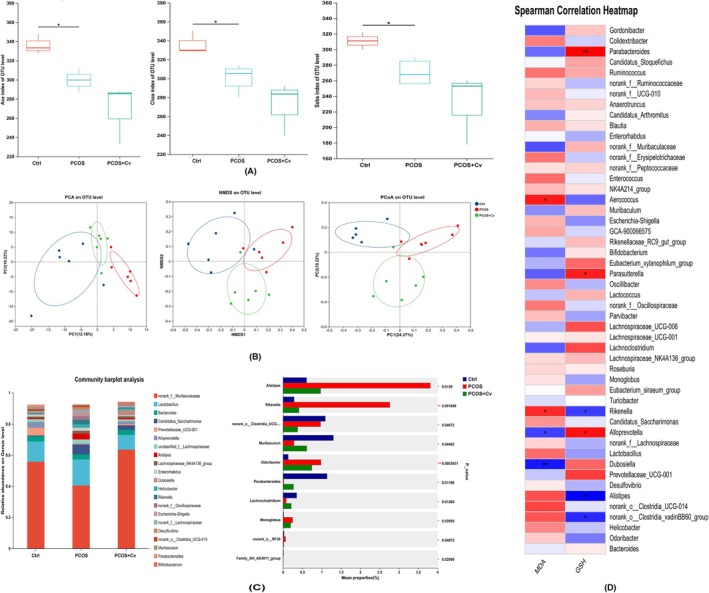
Gut microbiota profiling of mice by 16S rRNA gene sequencing. (A) α‐diversity analysis, (B) β‐diversity analysis, (C) species composition analysis, (D) Analysis of the association between oxidative stress and intestinal flora. One‐way ANOVA and Tukey's multiple comparison tests were used. Statistical significance is indicated: *p < 0.05. The exact sample size (*n* = 6) for each experimental group.

Fecal samples were collected from mice prior to euthanasia, and gut microbiota composition was analyzed using 16S rRNA sequencing. Alpha diversity analysis revealed significant differences in the ACE, Chao1, and Sobs indices between the PCOS and Ctrl groups (Figure 6A). Beta diversity analysis, including PCA, PCoA, and NMDS, demonstrated distinct clustering among the three groups, indicating notable differences in gut microbial composition (Figure 6B). At the genus level (Figure 6C), the PCOS group exhibited a significant decrease in the abundance of beneficial genera such as norank*_f__Muribaculaceae, Prevotellaceae_UCG‐001, Alloprevotella, Dubosiella, Parabacteroides, Candidatus_Saccharimonas, norank_f__Oscillospiraceae*, and *Escherichia‐Shigella*. In contrast, the abundance of certain pathogenic and inflammation‐related genera was increased.

Correlation analysis between gut microbiota and serum levels of GSH and MDA (Figure 6D) revealed that *Parasutterella*, *Alloprevotella*, *Dubosiella*, and *Parabacteroides* were positively correlated with GSH and negatively correlated with MDA, whereas *Rikenella* and *Alistipes* showed negative correlations with GSH and positive correlations with MDA. These results suggest that *Chlorella* plays an important role in modulating gut microbiota composition and alleviating dysbiosis in PCOS mice.

## 4 Discussion

In this study, we constructed a PCOS model using DHEA‐induced pubertal mice, and systematically investigated the regulatory effects of *Chlorella* on PCOS phenotype and intestinal flora. The experimental results showed that mice in the PCOS group developed typical endocrine disorders, including elevated testosterone and luteinizing hormone, as well as an imbalance of estradiol and abnormal ovarian morphology, characterized by a significant increase in ovarian coefficients and weights. These findings were consistent with previous studies and confirmed the validity of the model (Chen et al. 2025; Zhou et al. 2025). The DHEA‐induced PCOS mouse model showed a high similarity to human PCOS in terms of several key pathologic features: first, endocrine features both model mice and human patients showed hyperandrogenemia and gonadotropin imbalance (abnormal LH/FSH ratio), which are the core diagnostic indicators of PCOS (Gałczyńska et al. 2025; Jahan et al. 2025; Raihanah et al. 2025); second, at the level of ovarian pathology, follicular stagnation, polycystic changes, and decreased luteinization were observed in both mice and human patients (Xie et al. 2025); and third, metabolic abnormalities were observed in both mice and human patients, even though the metabolic disturbances in the model were weaker than those in human PCOS patients (Li et al. 2016). In terms of metabolic abnormalities, although the degree of metabolic disorders in the DHEA‐induced model alone was weaker than that in the clinical cases, phenotypes such as weight gain and insulin resistance trend were still observed in the model mice, which were common to metabolic syndromes in human PCOS patients (Kumar et al. 2025). However, the model also has limitations, such as the inability to fully mimic the polygenic genetic background (Ding et al. 2025; Tehrani et al. 2025) of human PCOS and the concomitant multi‐system dysfunction (increased cardiovascular risk) (Pirim and Bağcı 2025). In the future, the model can be combined with dietary interventions to further improve the fit between the model and the pathological features of the human disease.

In this study, we found that *Chlorella* intervention significantly reduced body weight and ovarian coefficients and improved endocrine disorders, with decreased testosterone levels and a modulated LH/FSH ratio, as well as ovarian pathological damage, including a decreased number of vesicles and an increased number of corpus luteum, in PCOS mice. This result is closely related to the antioxidant and anti‐inflammatory properties of *Chlorella*.

We further demonstrated that the improvement of ovarian function may be partially mediated by reduced oxidative stress. Evaluation of MDA and GSH levels in both serum and ovarian.

283 tissues showed that *Chlorella* supplementation significantly decreased MDA and increased GSH in both compartments. This dual‐level assessment confirms that systemic changes reflect ovarian oxidative stress status (Brizzi et al. 2025). Furthermore, we explored the NRF2/KEAP1 antioxidant pathway. *Chlorella* treatment reduced KEAP1 and increased NRF2 and GPX4 protein expression in ovarian tissues. To functionally validate this pathway, we constructed a PCOS‐like in vitro model using DHEA‐induced KGN cells and found that treatment with sulforaphane (SFN), a known NRF2 activator, significantly alleviated oxidative stress and modulated the expression of NRF2/KEAP1 pathway‐related genes. These results suggest that NRF2/KEAP1 plays a key functional role in *Chlorella*‐mediated antioxidant effects.

It is worth noting that there may be a multi‐target effect of endocrine regulation by *Chlorella*. On the one hand, by improving the oxidative stress state of ovarian granulosa cells, *Chlorella* may promote the activity of aromatase (CYP19A1), which promotes the conversion of androgen to estrogen (Chaudhary et al. 2025), thus reducing the testosterone level; on the other hand, it may indirectly regulate the function of the hypothalamus‐pituitary‐ovary axis (HPO axis), which can normalize the disordered LH/FSH ratio (Lonardo et al. 2024; Schniewind et al. 2021; Xu 2023). This finding is consistent with previous studies showing that natural products improve PCOS through multiple pathways (Atta et al. 2025; Usatiuc et al. 2025), suggesting that *Chlorella* has potential application in PCOS treatment.

*Chlorella* intervention significantly remodeled the gut microbiota structure in PCOS mice. We observed that *Chlorella* increased the abundance of beneficial bacteria. For instance, *Rikenella*, a genus known for its potential beneficial effects on gut health and metabolic regulation, became more abundant (Ma, Wu, et al. 2025; Ma, Chen, et al. 2025). At the same time, *Chlorella* decreased the proportion of pathogenic bacteria. *Helicobacter*, recognized for its association with gastrointestinal diseases (Ayala et al. 2025), and *Alistipes*, which has been linked to various metabolic disorders (Lu et al. 2025), both exhibited a reduction in their relative abundances. This change in the gut microbiota may be attributed to the dietary fiber and polysaccharide‐rich components of *Chlorella*. These components can be fermented as prebiotics by intestinal bacteria, leading to the production of short‐chain fatty acids (SCFAs). Prominent examples of these SCFAs are butyric acid and propionic acid, which potentially contribute to the overall beneficial effects of *Chlorella* on PCOS mice (Bañares et al. 2025; Cabrita et al. 2023). These metabolites not only regulate the intestinal barrier function, but also influence host metabolism and immune status through the “gut‐ovary axis” (da Silva et al. 2024; Geng et al. 2025). Although the present study did not directly verify the specific signaling pathway of the “gut–ovary axis”, the correlation analysis between the changes in microbial community structure and the indicators of oxidative stress provides important clues to reveal the potential mechanism of *Chlorella* to improve PCOS by regulating the gut microbiota.

## 5 Conclusion

*Chlorella* supplementation effectively alleviated the pathological features of PCOS in mice, including excessive body weight, ovarian dysfunction, hormonal imbalances, and oxidative stress. Moreover, it significantly reshaped the intestinal microbiota, suggesting a potential link between gut health and ovarian function. These findings offer novel theoretical insights into the use of *Chlorella* as a promising adjunctive strategy for the prevention and management of PCOS and its related complications.

## Author Contributions

**Yu Sun:** conceptualization (lead), data curation (lead), formal analysis (lead), investigation (lead), writing – original draft (lead). **Yanan Wang:** writing – review and editing (equal). **Jiaojiao Qi:** data curation (supporting), formal analysis (supporting), investigation (supporting). **Ruipeng Gao:** investigation (supporting), methodology (supporting). **Suxu Tan:** supervision (equal), writing – review and editing (equal). **Shuang Wang:** data curation (supporting), investigation (supporting). **Qing Zhang:** conceptualization (supporting), data curation (supporting), methodology (supporting). **Xuelin Gong:** conceptualization (supporting), investigation (supporting). **Zhenxia Sha:** conceptualization (lead), supervision (supporting). **Shichao Xing:** conceptualization (lead), funding acquisition (lead), writing – original draft (supporting).

## Supporting information


**Table S1:** Primer sequences.

## Data Availability

All data generated or analyzed during this study are included in this published article and (its Supporting Information). The datasets used and/or analyzed during the current study are available from the corresponding author on reasonable request.
