# Supplementary material for: The Effects of Chlorella vulgaris on Polycystic Ovary Syndrome in Mice
Source: Food Sci Nutr. 2025 Oct 15;13(10):e70992. doi: 10.1002/fsn3.70992 (PMC12522022; doi:10.1002/fsn3.70992)
Supplement: Supplementary file 1 — Table S1: Primer sequences. [file FSN3-13-e70992-s001.docx]

Table S1 Primer sequences

| **Derived from** | **Primer** | **Nucleotide Sequences（5’-3’）** |
| --- | --- | --- |
| Mice | STAR-qF | GGGCATACTCAACAACCAGGAAGG |
| Mice | STAR-qR | CTACCACCACCTCCAAGCGAAAC |
| Mice | CYP11A-qF | CACTCCTCAAAGCCAGCATCAAG |
| Mice | CYP11A-qR | ATTACGAAGCACCAGGTCATTCA |
| Mice | CYP19A-qF | TCAGCAAGTCCTCAAGCATGTTCC |
| Mice | CYP19A-qR | TTCTCCAAAGGCTCGGGTTGTT |
| Mice | KEAP1-qF | TGGTCGCCCTGTGCCTCTATG |
| Mice | KEAP1-qR | TGCCACTCGTCCCGCTCTG |
| Mice | NRF2-qF | GTTGCCACCGCCAGGACTAC |
| Mice | NRF2-qR | GTGCTCAGAAACCTCCTTCCAAAAC |
| Mice | GPX4-qF | GCAGGAGCCAGGAAGTAATCAAG |
| Mice | GPX4-qR | ACAGTGGGTGGGCATCGTC |
| Mice | GAPDH-qF | CACGGCAAATTCAACGGCACAG |
| Mice | GAPDH-qR | TCGCTCCTGGAAGATGGTGATGG |
| Human | KEAP1-qF | TGGAAAGAGCAGGCTTCCAG |
| Human | KEAP1-qR | CCCCTCCCAGGTATCCAAGA |
| Human | NRF2-qF | GGTTCCAAGTCCAGAAGCCA |
| Human | NRF2-qR | GGTTGGGGTCTTCTGTGGAG |
| Human | GAPDH-qF | CTGCCAACCGTGTCAGTGGTG |
| Human | GAPDH-qR | TCAGTGTAGCCCAGGATGCC |
